# Optimizing drip irrigation density enhances growth, yield, physiology, and antioxidant capacity of Korean strawberry ‘Kuemsil’ in recycling hydroponics

**DOI:** 10.7717/peerj.21637

**Published:** 2026-08-24

**Authors:** Youngae Jeong, Mewuleddeg Zebro, Minkyung Kim, Md Golam Rabbani, Ki-Young Choi

**Affiliations:** 1 College of Agriculture and Life Sciences, Department of Agriculture and Industries, Kangwon National University Graduate School, Chuncheon, Republic of South Korea; 2 Agriculture and Life Sciences Research Institute, Kangwon National University, Chuncheon, Republic of South Korea; 3 College of Agriculture and Veterinary Medicine, Department of Horticulture and Plant Sciences, Jimma University, Jimma, Ethiopia; 4 Department of Smart-Farm, U1 University, Yeongdong, Chungbuk, Republic of Korea; 5 College of Agriculture and Life Sciences, Department of Smart Farm and Agricultural Industry, Kangwon National University, Chuncheon, Republic of South Korea

**Keywords:** Dripper density, Vegetative growth, Water-use efficiency, Photosynthetic efficiency, Membrane stability, Antioxidant capacity

## Abstract

Efficient irrigation management is crucial for improving water-use efficiency and maintaining high productivity in controlled-environment strawberry cultivation. This study evaluated how different drip irrigation densities influence the growth, physiological responses, yield, fruit quality, mineral uptake, and antioxidant activity of the ‘Kuemsil’ strawberry cultivar grown in a recycling hydroponic system. Three irrigation densities, D3 (three drippers at 25 cm spacing), D6 (six drippers at 15 cm spacing), and D9 (nine drippers at 10 cm spacing) were compared under coir-substrate cultivation from October 2024 to May 2025. Increasing dripper density generally improved plant performance, with D6 (moderate) and D9 (high) irrigation densities promoting better vegetative growth, photosynthetic efficiency, water status, and nutrient balance compared with the D3 (low-density) treatment. Plants under low irrigation distribution showed reduced physiological performance and greater energy dissipation, indicating higher stress levels. Although the highest irrigation density increased total fruit production, the moderate irrigation density provided the best balance between marketable yield and fruit quality by reducing the proportion of unmarketable fruits and enhancing the production of larger fruits. In addition, the moderate irrigation density improved antioxidant capacity and membrane stability, suggesting enhanced physiological resilience under recycling hydroponic conditions. The findings demonstrate that optimizing drip irrigation density is critical for maximizing strawberry productivity and fruit quality while supporting efficient water and nutrient use in sustainable hydroponic production systems. The moderate irrigation density was identified as the most effective strategy for achieving balanced growth, yield, and physiological performance in ‘Kuemsil’ strawberry cultivation.

## Introduction

Water is critical for crop production, and efficient management is essential for meeting plant requirements, conserving resources, and avoiding stress-related losses. In controlled environments, irrigation is a key determinant of yield and quality. Both insufficient and excessive water supply can cause physiological disorders and significantly reduce fruit quality (Zhou et al., 2023). Innovative systems like hydroponics have emerged as resource-efficient alternatives to conventional soil-based cultivation, delivering water and nutrients directly to the root zone (Dutta, Mukherjee & Sawarkar, 2023). Recycling hydroponic systems enhance efficiency by reusing nutrient solutions, reducing water use and nutrient runoff, and lowering environmental impact (Asaduzzaman, Niu & Asao, 2022). These systems are particularly suitable for high-value crops like strawberries, where precise control over water and nutrient delivery can enhance quality and profitability.

Water availability is directly linked to nutrient uptake and transport. Inadequate irrigation reduces root function and solute accumulation in the root zone, hindering the absorption of essential nutrients (Mao et al., 2022). Conversely, excessive irrigation can dilute nutrient concentrations, compromising plant nutrition. These imbalances significantly impact fruit quality traits such as soluble solids content (SSC), titratable acidity, vitamin C content, and antioxidant compounds like anthocyanins and phenolics (Kim et al., 2022). However, over-irrigation often results in diluted sugars, softer fruit texture, and decreased marketability due to shorter shelf life.

Irrigation levels play a critical role in regulating the physiological and biochemical performance of strawberry plants in hydroponic cultivation. Adequate water maintains turgor pressure, promotes stomatal opening, and facilitates gas exchange for optimal photosynthesis (Yi, Chen & Anderson, 2022). Well-irrigated plants show higher chlorophyll content, enhanced photosynthetic activity, and improved metabolic performance. Insufficient irrigation causes stomatal closure to reduce water loss, limiting CO₂ assimilation and photosynthetic efficiency (Blanke & Cooke, 2004). Excessive irrigation can create hypoxic conditions in the root zone, impairing nutrient uptake and photosynthesis (Daniel & Hartman, 2024). Water use efficiency (WUE) reflects the balance between carbon assimilation and water consumption, with moderate water limitation sometimes enhancing WUE, while severe stress suppresses photosynthesis and transpiration (Zhao et al., 2020). Relative water content (RWC), an indicator of plant water status, declines under water deficit, leading to reduced gas exchange, restricted growth, and lower biomass, ultimately affecting fruit yield and quality. Irrigation regimes also influence biochemical stress responses in strawberry plants. Water deficit stimulates reactive oxygen species (ROS) production, causing oxidative stress and activating antioxidant defense systems like superoxide dismutase (SOD), catalase (CAT), and peroxidases (POD). Osmoprotective compounds such as proline and soluble sugars accumulate to maintain osmotic balance and membrane stability (Sun et al., 2015). Prolonged stress increases malondialdehyde (MDA) accumulation, indicating lipid peroxidation in membranes and cellular damage (Zahedi et al., 2023). While these responses are well documented under conventional irrigation systems, limited information exists on how irrigation distribution density in recirculating hydroponic systems affects the physiological, water relations, and antioxidant responses of strawberry plants.

In spite of these insights, the evaluation of the effects of varying levels of drip irrigation on specific strawberry cultivars, particularly under recycling hydroponic conditions, is limited. Most existing research emphasizes soil-based cultivation, with little focus on how different irrigation strategies affect morphological, physiological, and biochemical responses in controlled environments. The ‘Kuemsil’ strawberry cultivar, developed in South Korea and known for its favorable yield potential and fruit quality, has not been extensively studied in this context. Understanding its response to varying levels of drip irrigation in a recycling hydroponic system is crucial for optimizing production. Therefore, this study aims to investigate how varying drip irrigation levels affect the morphological, physiological, and biochemical characteristics of the ‘Kuemsil’ strawberry cultivar grown in a recycling hydroponic system. The experiment seeks to enhance water use efficiency, maximize crop productivity, and improve fruit quality. Ultimately, the findings will contribute to the development of more sustainable and resource-efficient smart farming practices for strawberry cultivation.

## Materials and Methods

### Plant material and experimental conditions

This study was conducted in a climate-controlled Venlo-type smart glasshouse at Kangwon National University in Chuncheon, South Korea. The plant material used was the ‘Kuemsil’ strawberry (*Fragaria × ananassa*) cultivar, developed by the Gyeongsangnam-do Agricultural Research and Extension Services, Korea. Seedlings used for this study were sourced from a commercial strawberry farm in PyeongChang, South Korea. At the time of transplanting, the seedlings had an average petiole length of 18.5 cm, leaf length of 9.8 cm, leaf width of 7.2 cm, and crown diameter of 10.5 mm. Transplanting was carried out on September 24, 2024, into 100% dust coconut coir slab (L100 × W20 × H15 cm; Duck Yang Coco, Sri Lanka). Each slab contained twelve plants, with six on each side. For 4 weeks before the experiment, seedlings were acclimated to the greenhouse environment using one dripper for every two plants, resulting in six drippers per slab. The first fruit harvest began on December 19, 2024. Thereafter, fruit thinning and removal of old leaves were performed according to general cultivation practices. Environmental conditions were monitored and controlled using a Ridder CX500 climate control system (Ridder HortiMaX, Harderwijk, The Netherlands). During the growing period, temperatures ranged from 11.2 °C to 25.8 °C, with an average of 16.3 °C. Relative humidity varied between 55.0% and 89.6%, averaging 76.2%. Solar radiation ranged from 801 to 1,955 J·cm^−2^, with an average of 1,257 J·cm^−2^.

**Figure 1:**
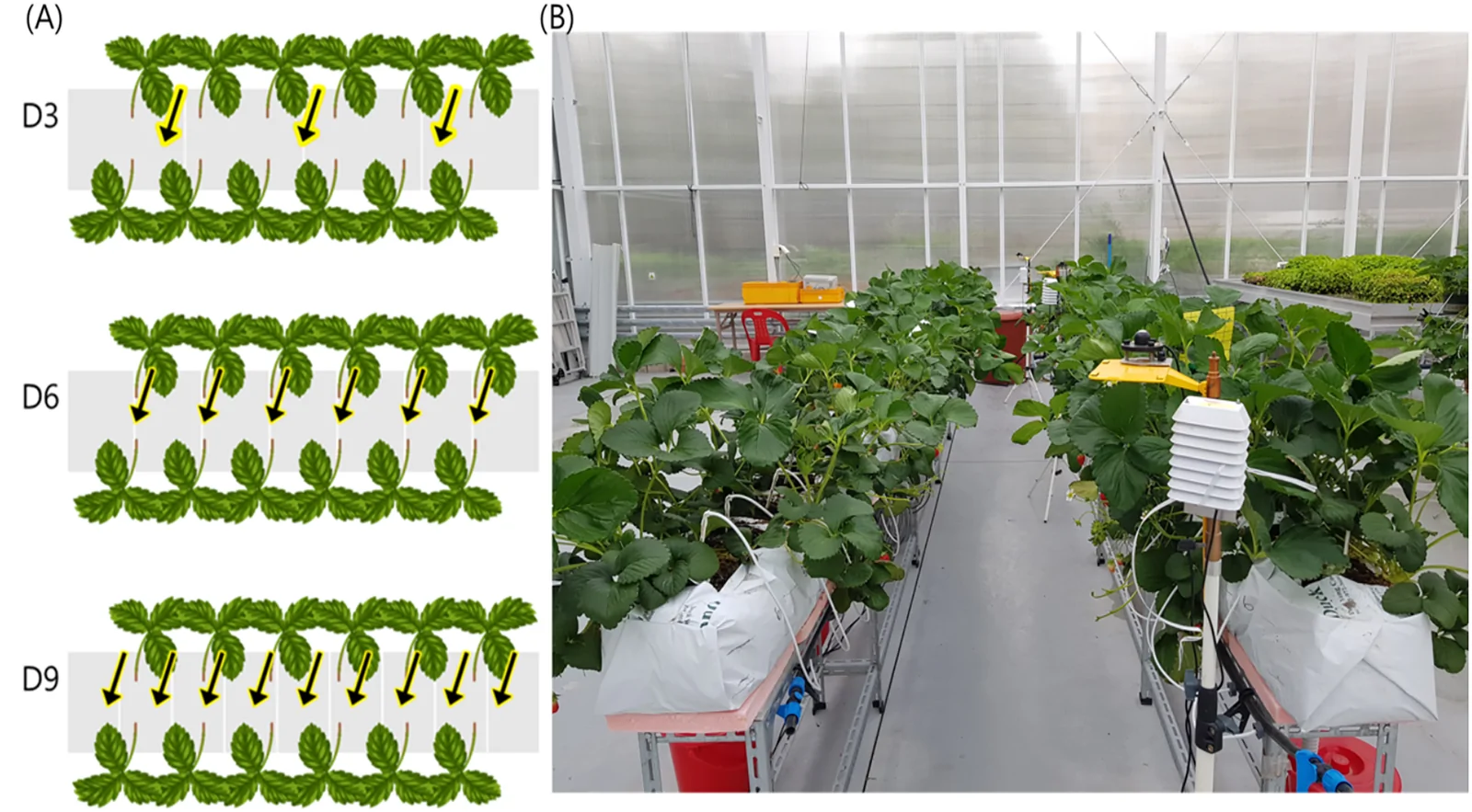
Experimental setup of the drip-density treatments and greenhouse arrangement of the strawberry cultivation system. (A) Schematic illustration of the three drip-density treatments, showing the number and distribution of drippers per cultivation slab. Treatments consisted of three drippers (D3), six drippers (D6), and nine drippers (D9) per slab, as indicated by the arrows. (B) Greenhouse layout of the experiment, showing strawberry plants grown in substrate-filled cultivation slabs arranged in two replicated blocks.

**Figure 2:**
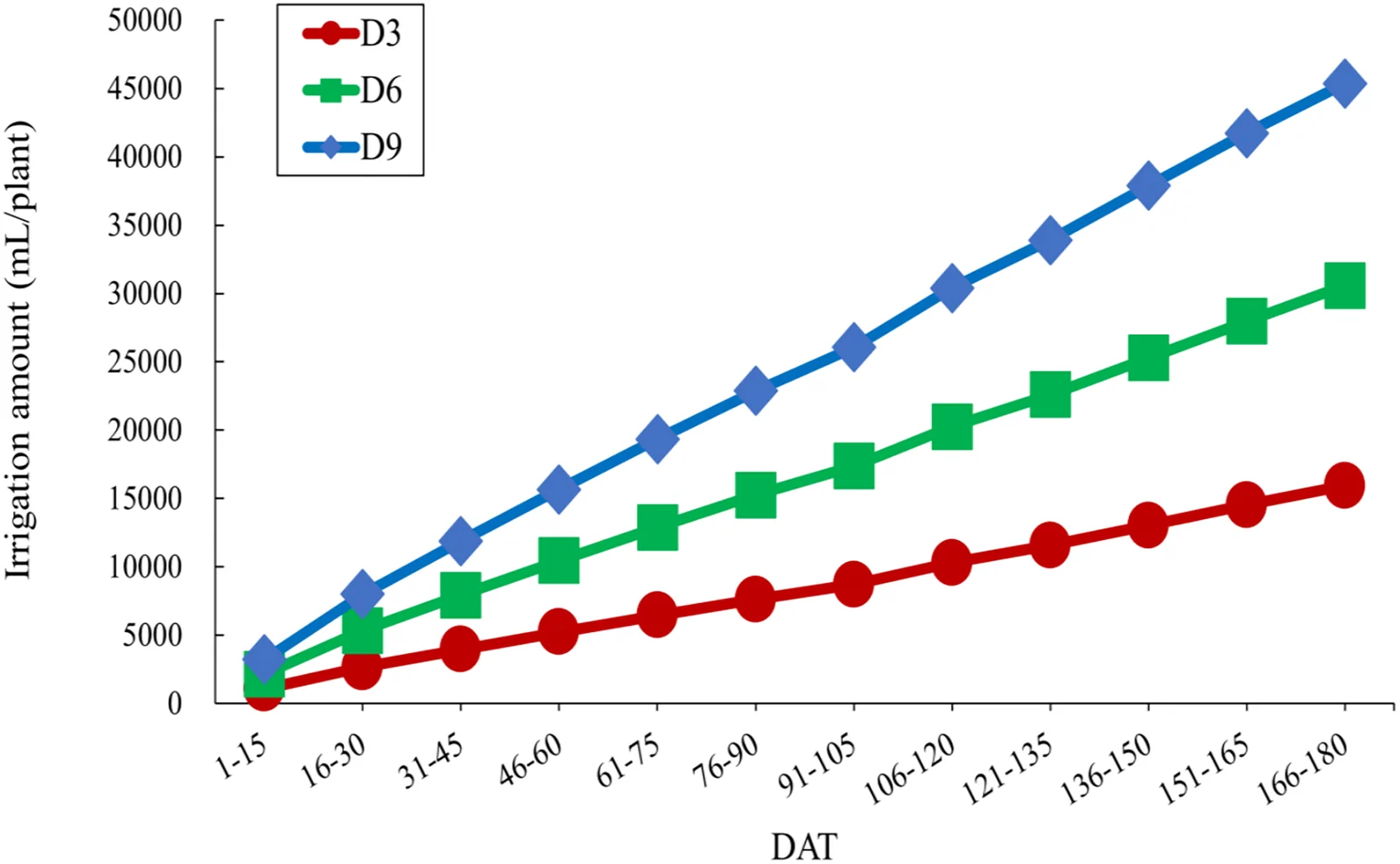
Cumulative irrigation volume applied throughout the experimental period, illustrating the total amount of water delivered from the initiation to the termination of the irrigation treatments.

The experiment was conducted from 22 October 2024 to 20 May 2025 to evaluate the effects of different drip irrigation levels on strawberry growth and development in a recycling hydroponic system. Three drip irrigation treatments were established based on the number and spacing of drippers: D3 (three drippers spaced 25 cm apart), D6 (six drippers spaced 15 cm apart), and D9 (nine drippers spaced 10 cm apart). Each treatment consisted of two slab replicates, with 12 plants per replicate. The overall experiment setup is shown in Fig. 1. Plants were supplied with the Japan Horticultural Research Institution nutrient solution (N–P–K–Ca–Mg = 17.3–4–8–8–4 me·L^−1^). In the recycling hydroponic system, the nutrient solution consisted of 60–70% drainage solution mixed with 30–40% fresh nutrient solution. The average concentrations of ions in the nutrient solution (ppm) were Na 58.8, K 168.8, NH_4_^+^-N 5.0, Ca 55.7, Mg 14.9, Cl 74.9, NO_3_^−^-N 381.7, P 23.5, and SO_4_ 124.3. The electrical conductivity (EC) and pH of the nutrient solution were maintained at 1.2 dS·m^−1^ and 5.5–6.5, respectively. Irrigation volume was adjusted according to daily weather conditions: 320 mL per dripper on sunny days and 50% of that volume on cloudy days. During the treatment period, the average daily irrigation volumes were 40–90 mL·plant^−1^ for D3, 80–160 mL·plant^−1^ for D6, and 160–240 mL·plant^−1^ for D9. Irrigation was applied for 1 min every 30 min between 09:00 and 14:00, resulting in a total irrigation time of 11 min per day, with a frequency of 5–11 irrigation events per day depending on environmental conditions. The cumulative irrigation volume during the experimental period is presented in Fig. 2.

### Drainage related parameters

Drainage volume was measured daily using a measuring cylinder, with values expressed in mL·plant^−1^·day^−1^. Drainage rates (%) and retained volume (mL·plant^−1^·day^−1^) were calculated using the formula below. Drainage electrical conductivity (EC) and pH were measured with a portable EC/pH meter (HI 9814; HANNA Instruments, Inc., Woonsocket, RI, USA).

${\rm Drainage\; rate} = \displaystyle{{{\rm Drainage\; amount\; }} \over {{\rm Irrigation\; amount}}} \times 100$

${\rm Retained\; volume} = {\rm irrigation\; volume} - {\rm drainage\; volume}$

### Growth and fruit characteristics measurements

Fruit-related parameters were assessed from December 19, 2024, to March 31, 2025 (58 to 160 days after treatment, DAT), and the average values are presented in this study. The fruits were harvested once or, in some cases, twice per week depending on their maturity level (≥90% ripeness). Fruit length and width were measured with a digital caliper, while fruit weight was recorded using an analytical balance (HS5200S; Hansang Instrument Co. Ltd., Gwangmyeong-si, Korea). Fruits weighing less than 10 grams were considered unmarketable, and their number and weight were recorded separately. Fruit firmness was determined using firmness tester (FR-5105; Lutron Electronic 140 Enterprise Co., Ltd., Taipei City, Taiwan). Soluble solids content (SSC) was measured using a digital refractometer (PAL-1; Atago, Tokyo, Japan) and expressed in degrees Brix (°Brix), while titratable acidity (TA) was determined with a digital acidity meter (PAL-Easy Acidity, Atago, Tokyo, Japan) and expressed as a percentage. Both SSC and TA were evaluated three time per month. Plant height, petiole length, leaf length, and leaf width were measured using a ruler, with values expressed in centimeters (cm). The number of leaves per plant was counted manually, whereas crown diameter was measured at its widest point using a digital caliper. Fresh weight was recorded immediately after harvest using a calibrated analytical balance (PAG4102; Ohaus Corporation, Parsippany, NJ, USA) with a precision of 0.01 grams. Dry weight was measured using the same balance after the plant tissues were oven-dried at 80 °C for 72 h. These growth-related parameters were recorded on May 20, 2025 (210 days after treatment).

Water use efficiency of the fruit in the recycling system was calculated using the following equation.

${\rm WUE} = \displaystyle{{{\rm Fruit\; yield\; }} \over {{\rm Irrigation\; amount} - {\rm drainage\; amount}}}$

### Leaf relative water content and electrolyte leakage measurements

Leaf relative water content (LRWC) and electrolyte leakage (EL) of strawberry plants were measured on April 30, 2025 (190 DAT). To evaluate LRWC, leaf samples were collected, and their fresh weight (FW) was immediately recorded. The leaves were then placed in tubes containing distilled water and kept at room temperature for 24 h. Then after, the surface moisture was gently removed, and the turgid weight (TW) was measured. The samples were subsequently dried in an oven to obtain the dry weight (DW). LRWC was calculated using the following formula:

${\rm LRWC} = \displaystyle{{{\rm FW} - {\rm DW\; }} \over {{\rm TW} - {\rm DW}}} \times 100$

To measure EL, 1 g of leaf tissue per replication was used to prepare leaf discs. The discs were placed in volumetric flasks containing 20 mL of ultra-pure water and incubated on a shaker for 12 h. Initial electrolyte leakage (ELi) was measured using a conductivity meter (DS-71G; HORIBA Ltd., Kyoto, Japan). The samples were then autoclaved at 121 °C for 15 min to release all electrolytes, and total electrolyte leakage (ELt) was recorded. EL was calculated using the following formula:

$${\rm EL\; }\left( {\rm \% } \right) = \displaystyle{{{\rm ELi}} \over {{\rm ELt}}} \times 100$$

### Leaf gas exchange and chlorophyll fluorescence measurements

Net photosynthetic rate, stomatal conductance, respiration rate, and intercellular CO₂ concentration were measured using a portable photosynthesis system (LI-6800; LI-COR Biosciences, Lincoln, NE, USA) on December 30, 2024, and April 20, 2025 (70 and 180 DAT, respectively). All measurements were conducted under controlled conditions: leaf temperature was maintained at 20 °C, reference CO_2_ concentration at 400 μmol mol^−1^, and photosynthetic photon flux density (PPFD) at 1,000 μmol m^−2^ s^−1^ and Relative humidity 60%. Chlorophyll fluorescence was assessed using a portable fluorometer (PAR-FluorPen FP 110/D; Photon Systems Instruments, Drásov, Czech Republic) on November 26, 2024 (35 DAT), February 24, 2025 (125 DAT), and April 30, 2025 (190 DAT). To ensure accurate fluorescence measurements, leaves were dark-adapted for 30 min before assessment.

### Chlorophyll and carotenoid extraction and quantification

chlorophyll and carotenoids content in strawberry leaves was measured on April 30, 2025 (190 DAT). A 0.5 g sample of fresh leaf tissue was homogenized in 10 mL of 80% acetone and sonicated for 20 min. Then the homogenized samples were centrifuged at 5,000 rpm for 20 min at 4 °C using a LZ-1248R refrigerated centrifuge (Gyozen Co., Ltd., Gimpo, Korea). After centrifugation, the supernatant was collected, and its absorbance was measured at 663, 645, and 470 nm using a spectrophotometer to determine the concentrations of chlorophyll a, chlorophyll b, and carotenoids, respectively. The concentrations of these pigments were calculated using the following equations:

${\rm Chlorophyll\; a\; }\left( {{\rm mg}/{\rm g}} \right) = \displaystyle{{12.7\left( {{\rm A}663} \right) - 2.69{\rm \; }\left( {{\rm A}645} \right)*{\rm V}* {\rm D}} \over {1{\hbox{,}}000*{\rm W}}}$

${\rm Chlorophyll\; b\; }\left( {{\rm mg}/{\rm g}} \right) = \displaystyle{{22.9\left( {{\rm A}645} \right) - 4.68\left( {{\rm A}663} \right)*{\rm V}*{\rm D}} \over {1\hbox{,}000*{\rm W}}}$

${\rm Total\; chlorophyll\; }\left( {{\rm mg}/{\rm g}} \right) = \displaystyle{{20.2\left( {{\rm A}645} \right) + 8.02{\rm \; }\left( {{\rm A}663} \right){\rm *\; V*\; D}} \over {1\hbox{,}000}*{\rm W}}$

${\rm Carotenoid\; contents\; }\left( {{\rm mg}/{\rm g}} \right) = \displaystyle{{\left[ {1\textrm,000\left( {{\rm A}470} \right) + 3.27{\rm \; }\left\{ {\left( {{\rm chlorophyll\; a}} \right) - \left( {{\rm chlorophyll\; b}} \right)} \right\}} \right]*{\rm V}*{\rm D}} \over {{\rm W}*1\textrm,000*229}}.$

where V represents the volume of acetone used, D is the dilution factor, and W denotes the weight of the leaf sample used.

### Antioxidant property measurements

Leaf samples were collected on April 30, 2025, and dried in an oven at 70 °C for 48 h to analyze antioxidant properties. The dried samples were then ground into a fine powder. Next, 0.1 g of the powder was mixed with 7 mL of 100% methanol and homogenized using a vortex mixer. The mixture was centrifuged at 4,500 rpm for 20 min. A second extraction was performed using 3 mL of 80% methanol. Both extracts were combined together and kept in −20 °C for further analysis. Total phenolic content (TPC) was measured using the Folin–Ciocalteu method as described by Zebro & Heo (2024a). Briefly, 100 μL of leaf extract was mixed with 2.9 mL of Folin–Ciocalteu reagent. Then, 0.75 mL of 20% (w/v) sodium bicarbonate solution and 1.25 mL of distilled water were added. The sample was mixed and incubated at room temperature in the dark for 30 min. After incubation, the absorbance was recorded at 765 nm using a UV–Vis spectrophotometer (UV-1800; Shimadzu, Kyoto, Japan). Total flavonoid content (TFC) was analyzed using the method developed by Chang et al. (2002). Leaf extract (100 µL) was mixed with 1.5 mL methanol, 100 µL 1 M sodium acetate, 100 µL 10% AlCl_3_, and 2.9 mL distilled water, then incubated for 30 min in the dark at room temperature. Absorbance was measured at 415 nm, and the values was as mg quercetin equivalents per gram dry weight.

Antioxidant capacity was determined using the DPPH (2,2-diphenyl-1-picrylhydrazyl) assay and the ferric reducing antioxidant power (FRAP) assay. For the DPPH assay, the procedure described by Brand-Williams, Cuvelier & Berset (1995) was followed with minor modifications. A volume of 100 µL of leaf extract was combined with 2.9 mL of a 0.1 mM DPPH solution prepared in methanol. The reaction mixture was kept in the dark at room temperature for 30 min, after which absorbance was measured at 517 nm using a spectrophotometer. The FRAP assay was carried out based on the method reported by Zebro et al. (2025). The FRAP reagent was freshly prepared by mixing 10 mM TPTZ in 40 mM HCl, 300 mM acetate buffer, and 20 mM FeCl_3_ at a volume ratio of 2.5:2.5:5 (v/v/v). For analysis, 20 µL of fruit extract was added to 2.98 mL of the FRAP reagent, and the mixture was incubated at 37 °C for 4 min and absorbance was recorded at 593 nm.

### Antioxidant enzyme activity

To evaluate the activities of antioxidant enzymes including SOD, CAT, and POD, we collected the youngest fully expanded leaves from each treatment group on April 30, 2025 (190 DAT). Immediately after collection, the leaf samples were flash-frozen in liquid nitrogen and stored at –80 °C until further analysis. The enzymatic activities of SOD, CAT, and POD were quantified using the method described by Zebro & Heo (2024b). SOD activity was determined by mixing 200 μL of the crude enzyme extract with 2 mL of reaction mixture containing 100 mM phosphate buffer (PBS, pH 7.8), 1 mM EDTA-2Na, 130 mM methionine, 750 μM nitro blue tetrazolium (NBT), and 20 μM riboflavin. CAT activity was measured by adding 200 μL of the crude enzyme extract into a cuvette, followed by 2 mL of reaction solution containing 100 mM PBS (pH 7.0) and 30% H_2_O_2_. The change in absorbance was recorded at 240 nm at 15-s intervals for 75 s. POD activity was assayed by mixing 200 μL of the crude enzyme extract with 2 mL of reaction solution containing 0.2% guaiacol, 100 mM PBS (pH 7.0), and 30% H_2_O_2_. The increase in absorbance was monitored at 470 nm every 15 s for 75 s.

### Evaluation of mineral contents

Leaf samples for mineral determination were collected on 20 May 2025 (210 days after transplanting, DAT). The samples were oven-dried and finely ground using a mortar and pestle. A 0.5 g aliquot of the powdered sample was placed into a glass flask, and 10 mL of 50% perchloric acid along with 1 mL of sulfuric acid were added. The mixture was thoroughly homogenized and allowed to stand overnight to complete the digestion reaction. The samples were then heated on a hot plate under a stepwise temperature program: 88 °C for 120 min, 180 °C for 30 min, and 350 °C for 120 min, until a clear solution was obtained. After digestion, the solutions were diluted 1,000-fold with distilled water and filtered sequentially through Whatman No. 6 filter paper (3 µm pore size) and a 0.45 µm syringe filter. Mineral concentrations were quantified using inductively coupled plasma optical emission spectrometry (ICP-OES; Integra Dual, GBC, Australia). Standard solutions for magnesium, sodium, and potassium were obtained from Kanto Chemical Co., Inc. (Tokyo, Japan), while calcium and phosphate standards were purchased from Samchun Chemical (Gyeonggi-do, Korea) and Supelco (Sigma-Aldrich, Darmstadt, Germany), respectively.

### Statistical analysis

The experiment was arranged in a randomized complete block design (RCBD) with two blocks to account for potential spatial variation within the greenhouse. Each treatment was applied to two cultivation slabs, with 12 plants grown per slab. Within each block, all treatments were represented by one slab, and the positions of the treatment slabs were randomly assigned. During statistical analysis, treatment was considered a fixed effect, whereas block was treated as a random effect. Statistical analyses were conducted to evaluate differences among treatments and to explore the associations between measured variables. Growth-related traits were assessed using plants, while fruit-related traits were analyzed with sixteen plants. Measurements of leaf antioxidant capacity were performed with six replicates. For the OJIP fluorescence parameters, leaf gas exchange traits, LRWC, EL, antioxidant enzyme activity and chlorophyll concentration, three replicates were utilized. The data were analyzed using SPSS software (version 26; IBM Corp., Armonk, NY, USA), and treatment means were separated using Duncan’s Multiple Range Test (DMRT) at a significance threshold of *p* < 0.05. Additionally, correlation analysis was conducted using SRplot (http://www.bioinformatics.com.cn/SRplot) and cluster analysis was conducted using MetaboAnalyst six software (https://www.metaboanalyst.ca/).

## Results

### Irrigation and drainage characteristics

Irrigation differences significantly affected drainage characteristics across various treatment periods (Table 1). From 1 to 60 DAT, drainage volume, retained volume, and drainage rate were significantly higher in D9, while D3 showed the lowest values. Higher drainage EC and lower pH levels were observed in D6 and D9. As plant growth stages progressed, drainage volume and rate decreased, while retained volume increased. Lower drainage volume and rate were recorded during the 121–180 DAT period compared to the 1–60 DAT and 61–120 DAT periods. At this DAT, the drainage rate of D3 was low (1.4%), indicating a non-drainage condition, and the RV increased by 20 mL. In contrast, RV increased by 35 mL in D6 and 53 mL in D9. In contrast, drainage EC increased, with D3 showing a significant rise during the 121–180 DAT period compared to earlier periods. Overall, drainage volume, retained volume, and drainage rate were consistently higher in D9, whereas lower values for these parameters were noted during the 61–120 and 121–180 DAT periods.

**Table 1:** Effects of different numbers of drippers per slab on the irrigation and drainage characteristics of strawberry plants from 1 to 180 days after treatment (DAT).

Treatments	Total irrigated volume (mL·plant^−1^·day^−1^)	Drainage volume (mL·plant^−1^·day^−1^)	Retained volume (mL·plant^−1^·day^−1^)	Drainage rate (%)	Drainage pH	Drainage EC (dS∙m^−1^)
1–60 DAT (October 23, 2024–December 21, 2024)						
D3	87.08 ± 18.0^cZ^	18.21 ± 16.9^c^	68.87 ± 15.9^c^	19.62 ± 16.2^b^	7.26 ± 3.0^a^	0.92 ± 0.4^b^
D6	174.15 ± 36.0^b^	98.08 ± 35.0^b^	87.14 ± 20.2^b^	48.88 ± 12.3^a^	6.98 ± 0.2^b^	1.22 ± 0.1^a^
D9	261.23 ± 54.0^a^	157.59 ± 50.8^a^	103.63 ± 23.0^a^	59.49 ± 9.2^a^	6.84 ± 0.2^b^	1.27 ± 0.1^a^
61–120 DAT (December 22, 2024–February 19, 2025)						
D3	81.13 ± 20.0^c^	12.68 ± 13.8^c^	71.78 ± 35.0^b^	14.40 ± 11.3^c^	7.17 ± 3.1^a^	1.56 ± 0.7^a^
D6	163.63 ± 79.2^b^	69.00 ± 28.7^b^	94.63 ± 62.7^ab^	42.80 ± 13.9^b^	6.91 ± 0.1^b^	1.58 ± 0.1^a^
D9	245.44 ± 118.8^a^	123.86 ± 24.5^a^	121.58 ± 111.1^a^	52.94 ± 11.6^a^	6.79 ± 0.1^b^	1.50 ± 0.1^a^
121–180 DAT (February 20, 2025–April 20, 2025)						
D3	94.04 ± 4.4^c^	1.03 ± 0.0^b^	93.01 ± 4.4^c^	1.41 ± 0.0^b^	7.17 ± 0.0^a^	1.90 ± 0.0^a^
D6	172.02 ± 5.2^b^	42.46 ± 21.5^a^	129.56 ± 24.3^b^	24.45 ± 12.8^a^	7.13 ± 0.2^a^	1.84 ± 0.1^a^
D9	249.19 ± 11.3^a^	73.19 ± 30.4^a^	175.99 ± 30.7^a^	29.34 ± 12.4^a^	7.03 ± 0.3^a^	1.68 ± 0.1^a^

**Note:**

Data are presented as mean ± SD (*n* = 60). ^Z^Different lowercase letters within the same column indicate significant differences among treatments according to Duncan’s multiple range test (DMRT) at *p* < 0.05. EC, electrical conductivity.

### Growth and fruit characteristics

**Figure 3:**
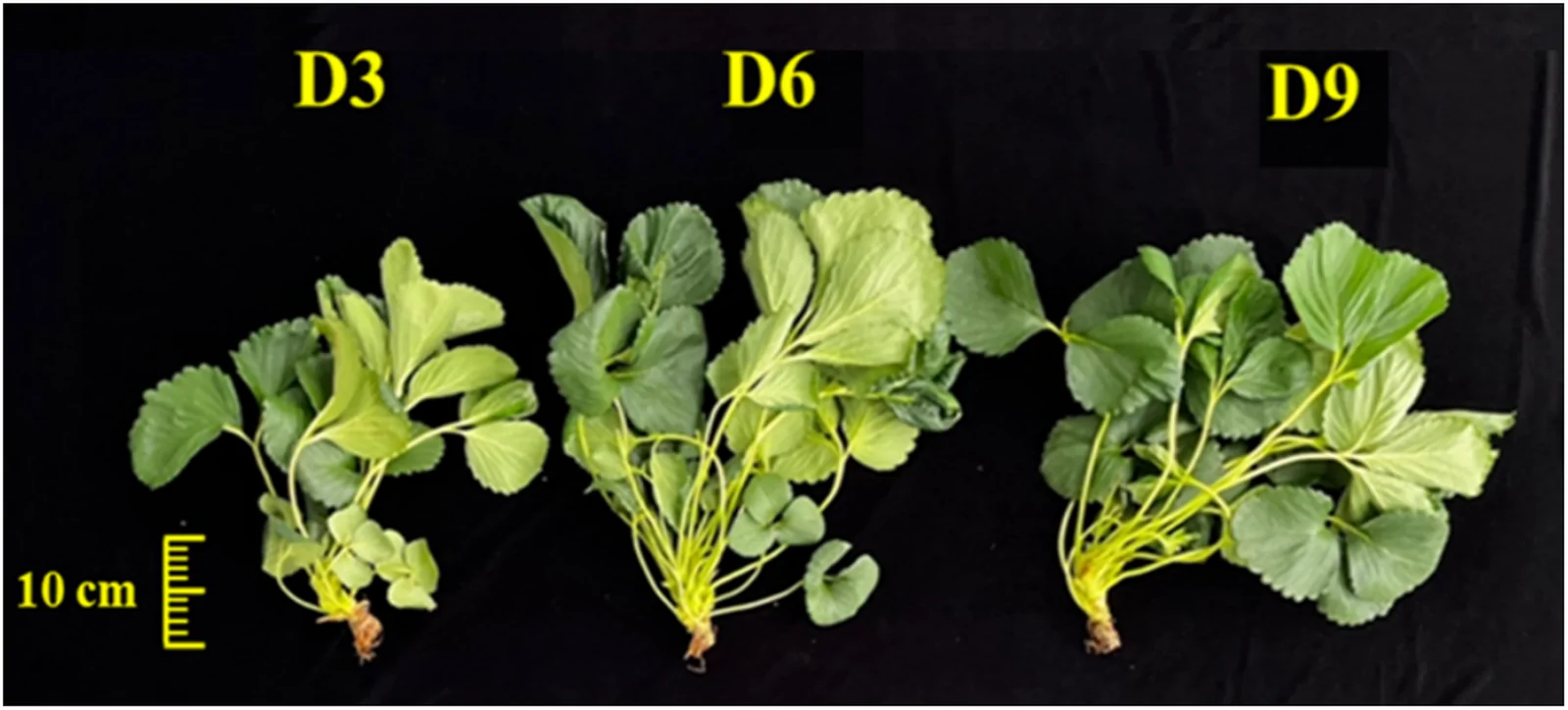
Effects number of drippers per slab on the growth performance of strawberry plants captured at the end of the treatments (May 20, 2025).

Table 2 shows the growth characteristics of strawberry plants under varying levels of drip irrigation conditions. Plants irrigated with D6 produced the highest number of leaves (19.1), which was significantly greater than those under D3. Additionally, the number of leaves in plants under D9 was statistically similar to that of D6. The height of the plants and the length of the petioles were also significantly influenced by the irrigation treatments. Both D6 and D9 resulted in taller plants, measuring 44.1 and 43.2 cm, respectively, and had longer petioles at 24.7 and 25.2 cm, compared to D3, which had a height of 36.7 cm and a petiole length of 21.1 cm (Table 2 and Fig. 3). Although leaf length, leaf width, and crown diameter did not show significant differences across the treatments, plants under D6 tended to have slightly larger values. However, the ratio of leaf length to width was significantly higher in D9 (1.30) compared to D3 and D6, which had ratios of 1.20 and 1.21, respectively. Total biomass production was highly influenced by the irrigation treatments; plants under D6 and D9 produced significantly higher shoot fresh weights of 126.4 and 123.3 g, respectively, compared to D3, which yielded 89.5 g. Similarly, dry weight accumulation was higher in D6 (27.7 g) and D9 (25.3 g) than in D3 (21.6 g), but there was no significant difference among the treatments.

**Table 2:** Effects of different numbers of drippers per slab on growth characteristics of strawberry plants at 210 DAT (May 20, 2025).

Treatments	Number of leaves	Plant heigh (cm)	Petiole length (cm)	Leaf length (cm)	Leaf width (cm)	Crown diameter (mm)	Leaf length/leaf width	Total fresh weight (g)	Total dry weight (g)
D3	17.6 ± 1.6^bZ^	36.7 ± 1.3^b^	21.1 ± 0.8^b^	12.0 ± 2.9^a^	10.0 ± 1.7^a^	17.8 ± 1.5^a^	1.20 ± 0.1^b^	89.5 ± 20.2^b^	21.6 ± 6.5^a^
D6	19.1 ± 0.9^a^	44.1 ± 1.6^a^	24.7 ± 1.6^a^	13.2 ± 2.2^a^	11.0 ± 3.2^a^	18.4 ± 1.7^a^	1.21 ± 0.1^b^	126.4 ± 1.0^a^	27.7 ± 6.5^a^
D9	18.8 ± 0.8^a^	43.2 ± 3.2^a^	25.2 ± 2.1^a^	13.0 ± 8.9^a^	9.9 ± 3.6^a^	18.1 ± 1.6^a^	1.30 ± 0.1^a^	122.9 ± 8.3^a^	25.6 ± 5.1^a^

**Note:**

Data are presented as mean ± SD (*n* = 10). ^Z^Different lowercase letters within the same column indicate significant differences among treatments according to DMRT at *p* < 0.05.

Furthermore, a significant difference in fruit quality attributes was observed among the treatments (Table 3). D6 produced the longest fruits (45.9 mm), which was significantly longer than those from D3 (42.8 mm) and slightly longer than D9 (44.8 mm), although the difference with D9 was not statistically significant. A similar trend was observed for fruit width, with D6 yielding the widest fruits (36.4 mm), significantly wider than D3 (34.1 mm), while D9 (35.2 mm) showed no significant difference. Fruit firmness did not vary significantly among the treatments; however, D3 fruits were slightly firmer (2.47 N/Ø3) compared to D6 (2.27 N/Ø3) and D9 (2.44 N/Ø3). The SSC ranged from 13.9 to 14.3 °Brix, with D6 having the highest value (14.3 °Brix), followed by D3 (14.0 °Brix) and D9 (13.9 °Brix). TA showed significant differences, with D3 having the highest level (0.76%), followed by D6 (0.69%) and D9 (0.66%). As a result, the SSC/TA ratio, which indicates perceived sweetness, was significantly higher in D9 (22.1) and D6 (21.4) compared to D3 (18.8), despite similar SSC levels.

**Table 3:** Effects of different numbers of drippers per slab on the characteristics of strawberry fruits from 58 to 160 DAT (December 19, 2024, to March 31, 2025).

Treatments	FW (g/fruit)	FL (mm)	FW (mm)	FF (N/Ø3)	SSC (°Brix)	TA (%)	SSC/TA
D3	18.0 ± 1.4^bZ^	42.8 ± 3.9^b^	34.1 ± 2.4^b^	2.47 ± 0.3^a^	14.0 ± 1.7^a^	0.76 ± 0.1^a^	18.8 ± 2.3^b^
D6	21.1 ± 1.8^a^	45.9 ± 4.0^a^	36.4 ± 2.6^a^	2.27 ± 0.2	14.3 ± 1.3^a^	0.69 ± 0.1^b^	21.4 ± 2.6^a^
D9	19.5 ± 1.5^a^	44.8 ± 4.1^ab^	35.2 ± 2.8^ab^	2.44 ± 0.3^a^	13.9 ± 2.5^a^	0.66 ± 0.1^b^	22.1 ± 3.3^a^

**Note:**

Data are presented as mean ± SD (*n* = 16). ^Z^Different lowercase letters within the same column indicate significant differences among treatments according to DMRT at *p* < 0.05. FW, fruit weight; FL, fruit length; FW, fruit width; FF, fruit firmness; SSC, soluble solids content; TA, titratable acidity.

To further assess the impact of varying levels of drip irrigation, fruit yield, marketability and WUE were measured (Table 4). The number of marketable fruits per plant ranged from 12.7 to 17.4, with the lowest count in the D3 treatment and the highest in D9. D6 had the fewest unmarketable fruits, while D3 had the most. The weight of marketable fruit varied from 228.5 to 338.8 g per plant, again lowest in D3 and highest in D9. In terms of fruit size distribution, D9 produced the highest numbers of fruits weighing 10–20 and 20–30 g, while D3 and D6 had statically similar counts. Fruits over 30 g were most abundant in D6 and least in D3. In the weight categories, the heaviest fruits, weighing 10–20 and 20–30 g, were found in D9, whereas those in D3 were the lightest. Fruits greater than 30 g had the highest weight in D6 and the lowest in D3. The unmarketable rate was highest in D3, whereas D6 showed a significantly lower value. Regarding WUE, D3 and D6 (2.97 and 3.1 g·mL^−1^, respectively) had higher values, while D9 (2.59 g·mL^−1^ plant^−1^) showed a significantly lower value.

**Table 4:** Effects of different numbers of drippers per slab on strawberry yield-related parameters and water use efficiency (WUE).

Treatments	Fruit number (No/plant)				Fruit weight (g/plant)				Unmarketable fruit rate^Y^ (%)	WUE^x^ (g∙mL∙plant^-1^)
	10–20 g	20–30 g	>30 g	Total	10–20 g	20–30 g	>30 g	Total		
D3	7.8 ± 2.1^bZ^	4.6 ± 1.2^b^	0.3 ± 0.5^b^	12.7 ± 1.5^c^	109.1 ± 31.6^b^	109.9 ± 29.3^b^	9.6 ± 0.4^b^	228.5 ± 27.9^c^	26.8 ± 9.8^a^	2.97 ± 0.4^a^
D6	7.5 ± 2.3^b^	5.0 ± 1.7^b^	2.2 ± 1.2^a^	14.7 ± 1.0^b^	111.2 ± 34.4^b^	123.7 ± 41.6^ab^	73.2 ± 39.4^a^	308.10 ± 17.2^b^	13.3 ± 9.2^b^	3.10 ± 0.2^a^
D9	9.9 ± 2.0^a^	5.9 ± 1.6^a^	1.6 ± 1.4^a^	17.4 ± 1.5^a^	145.0 ± 30.2^a^	142.1 ± 40.4^a^	51.6 ± 44.6^a^	338.8 ± 40.6^a^	15.7 ± 8.1^a^	2.59 ± 0.4^b^

**Note:**

Data are presented as mean ± SD (*n* = 16). Yield related parameters were measured from 58 to 160 DAT (December 19, 2024, to March 31, 2025). ^Z^Different lowercase letters within the same column indicate significant differences among treatments according to DMRT at *p* < 0.05. ^Y^Calculated as the number of unmarketable fruits divided by the total number of fruits. ^X^Calculated as total yield divided by retained volume.

### Physiological characteristics

**Figure 4:**
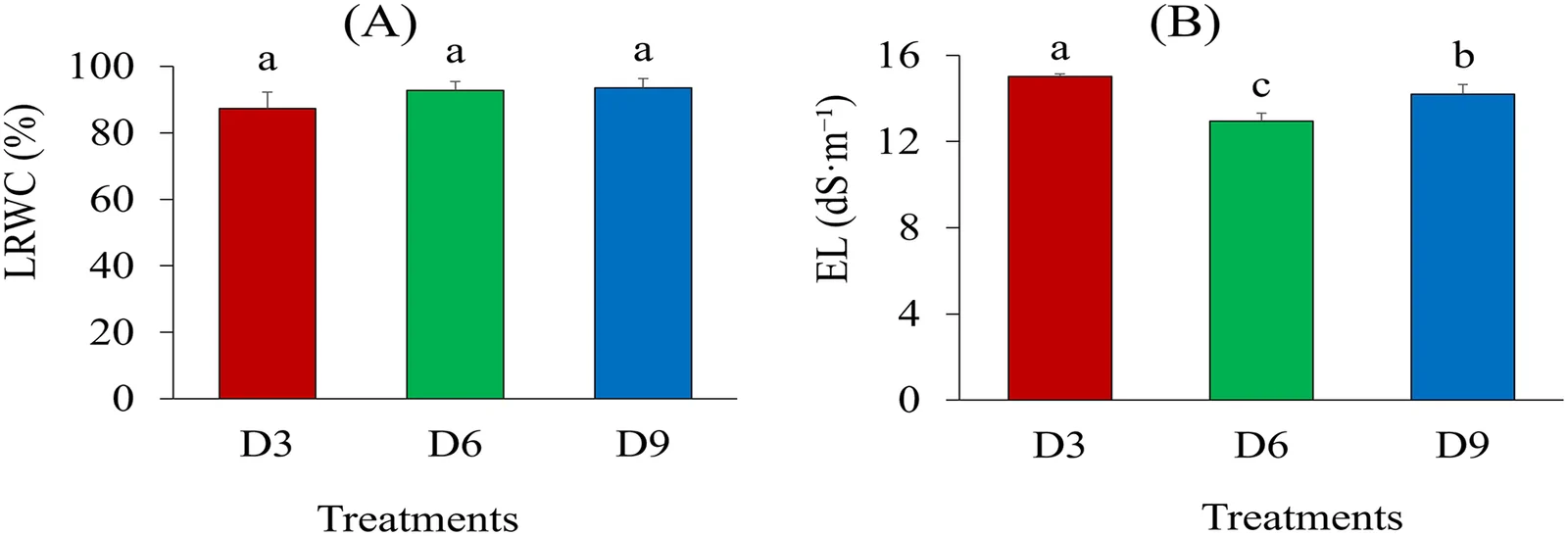
Effects of different numbers of drippers per slab on (A) leaf relative water content (LRWC) and (B) electrolyte leakage (EL) in strawberry plants at 190 DAT (April 30, 2025). Error bars represent the standard deviation (*n* = 3). Different letters above the bars indicate significant differences among treatments according to Duncan’s multiple range test (DMRT) at *p* < 0.05.

No significant difference was found in LRWC among the treatments (Fig. 4A). However, treatments D6 and D9 showed numerically higher LRWC values, while D3 had the lowest. In terms of EL, significant differences were observed among the treatments. Plants grown under the D6 treatment exhibited the lowest EL value (12.95 dS·m^−1^), while the highest was recorded in the D3 treatment (15.03 dS·m^−1^) (Fig. 4B). The D9 treatment had a value lower than D3 but higher than D6 (Fig. 4B).

Varying levels of drip irrigation also influenced the chlorophyll fluorescence parameters of strawberry leaves differently over time (Table 5). At 35 DAT, no significant differences were observed among the irrigation treatments for any of the parameters. The values of Fm/Fo (5.94–6.19), Fv/Fo (4.94–5.19), and Fv/Fm (0.83–0.84) remained similar across D3, D6, and D9. Additionally, PiAbs, ABS/RC, TRo/RC, ETo/RC, and DIo/RC showed no significant variation. At 125 DAT, the OJIP parameters continued to show no significant differences among the treatments. The ratios of Fm/Fo (5.40–5.59), Fv/Fo (4.40–4.59), Fv/Fm (0.81–0.82) and PiAbs (6.26–7.36) were comparable across treatments. The energy fluxes per reaction center (ABS/RC, TRo/RC, ETo/RC, and DIo/RC) remained consistent among treatments. By 190 DAT, clear differences emerged among the treatments. D3 showed the lowest values of Fm/Fo (4.56), Fv/Fo (3.55), and Fv/Fm (0.78), which were significantly lower than those of D6 (5.17, 4.17, 0.81, respectively) and D9 (5.14, 4.14, 0.80, respectively). Conversely, PiAbs did not differ significantly among treatments, although numerical values were lower in D3. Energy absorption and trapping per reaction center (ABS/RC and TRo/RC) were slightly higher in D3, but the most notable difference was in energy dissipation (DIo/RC), which was significantly higher in D3 (0.52) compared to D6 (0.43).

**Table 5:** Effects of different numbers of drippers per slab on chlorophyll fluorescence (OJIP) parameters of strawberry leaves at 35, 125, and 190 DAT.

Treatments	Fm/Fo	Fv/Fo	Fv/Fm	PiAbs	ABS/RC	TRo/RC	ETo/RC	DIo/RC
35 DAT (November 26, 2024)								
D3	6.16 **± **0.4^a^	5.16 **± **0.4^a^	0.84 **± **0.0093^a^	5.04 **± **1.1^a^	1.84 **± **0.11^a^	1.54 **± **0.07^a^	0.99 **± **0.02^a^	0.30 **± **0.04^a^
D6	5.94 **± **0.34^a^	4.94 **± **0.3^a^	0.83 **± **0.0085^a^	4.91 **± **0.1^a^	1.92 **± **0.05^a^	1.60 **± **0.04^a^	1.05 **± **0.03^a^	0.32 **± **0.02^a^
D9	6.19 **± **0.4^a^	5.19 **± **0.4^a^	0.84 **± **0.0098^a^	4.94 **± **0.9^a^	1.93 **± **0.03^a^	1.62 **± **0.02^a^	1.04 **± **0.05^a^	0.31 **± **0.02^a^
125 DAT (February 24, 2025)								
D3	5.52 **± **0.2^a^	4.52 **± **0.2^a^	0.82 **± **0.007^a^	6.26 **± **0.9^a^	1.85 **± **0.05^a^	1.51 **± **0.05^a^	1.07 **± **0.01^a^	0.34 **± **0.01^a^
D6	5.59 **± **0.1^a^	4.59 **± **0.1^a^	0.82 **± **0.0013^a^	6.49 **± **1.0^a^	1.84 **± **0.11^a^	1.50 **± **0.09^a^	1.04 **± **0.08^a^	0.34 **± **0.02^a^
D9	5.40 **± **0.1^a^	4.40 **± **0.1^a^	0.81 **± **0.0053^a^	7.36 **± **1.2^a^	1.70 **± **0.08^a^	1.38 **± **0.06^a^	0.98 **± **0.01^a^	0.32 **± **0.02^a^
190 DAT (April 30, 2025)								
D3	4.56 **± **0.3^bZ^	3.55 **± **0.3^b^	0.78 **± **0.0155^b^	2.52 **± **0.4^a^	2.36 **± **0.09^a^	1.84 **± **0.04^a^	1.14 **± **0.01^a^	0.52 **± **0.06^a^
D6	5.17 **± **0.3^a^	4.17 **± **0.3^a^	0.81 **± **0.0132^a^	3.87 **± **1.1^a^	2.19 **± **0.14^a^	1.75 **± **0.10^a^	1.15 **± **0.04^a^	0.43 **± **0.04^b^
D9	5.14 **± **0.4^ab^	4.14 **± **0.4^ab^	0.80 **± **0.0134^a^	3.42 **± **0.8^a^	2.25 **± **0.11^a^	1.80 **± **0.06^a^	1.16 **± **0.01^a^	0.44 **± **0.05^ab^

**Note:**

Data are presented as mean ± SD (*n* = 3). ^Z^Different lowercase letters within the same column indicate significant differences among treatments according to DMRT at *p* < 0.05. Fm/Fo, ratio of maximum to minimum fluorescence; Fv/Fo, ratio of variable to minimum fluorescence; Fv/Fm, maximum quantum efficiency of PSII; PIabs, performance index on absorption basis; ABS/RC, absorbed energy per reaction center; TRo/RC, trapped energy per reaction center; ETo/RC, electron transport per reaction center; DIo/RC, energy dissipation per reaction center.

Moreover, leaf gas exchange parameters varied significantly among treatments, particularly during the early growth stage (Table 6). At 70 DAT, the number of drippers had a strong influence on leaf gas exchange. Plants irrigated with D6 recorded the highest values for photosynthetic rate (Pn: 14.62 µmol m^−2^ s^−1^), stomatal conductance (Gs: 0.16 mol m^−2^ s^−1^), intercellular CO₂ concentration (Ci: 228 µmol mol^−1^), and transpiration rate (Tr: 1.55 mmol m^−2^ s^−1^). These values were significantly greater than those observed under D3, which showed the lowest Pn (12.68 µmol m^−2^ s^−1^), Gs (0.13 mol m^−2^ s^−1^), Ci (214 µmol mol^−1^), and Tr (1.28 mmol m^−2^ s^−1^). The D9 treatment showed statistically comparable values to D6 across all parameters. AT 180 DAT, although Pn, Gs, Ci, and Tr values were statistically similar among D3, D6, and D9, a numerical trend persisted, with slightly higher values under D6 (Pn: 14.54 µmol m^−2^ s^−1^; Ci: 230 µmol mol^−1^; Tr: 1.63 mmol m^−2^ s^−1^). The chlorophyll and carotenoid contents showed significant difference among the treatments (Table 7). The D6 treatment showed the highest levels of chlorophyll *a* (2.38 mg/g), chlorophyll *b* (1.06 mg/g), and total chlorophyll (3.45 mg/g), all significantly greater than those observed in the D3 treatment. Carotenoid content was also significantly highest in the D6 treatment (0.33 mg/g) compared to D3 treatment (0.26 mg/g), while the D9 treatment (0.30 mg/g) was statistically similar to both D3 and D6.

**Table 6:** Effects of different numbers of drippers per slab on leaf gas exchange parameters of strawberry plants at 70 and 180 DAT.

Treatments	Pn	Gs	Ci	Tr
	(µmol.CO_2_.m^−2^s^−1^)	(Mol m^−2^s^−1^)	(µmol mol^−1^)	(Mmol.H_2_O.m^−2^s^−1^)
70 DAT (December 30, 2024)				
D3	12.68 ± 2.4^bZ^	0.13 ± 0.033^b^	214 ± 20.2^a^	1.28 ± 0.4^b^
D6	14.62 ± 1.141^a^	0.16 ± 0.001^a^	228 ± 6.8^a^	1.55 ± 0.1^a^
D9	13.63 ± 1.2^a^	0.15 ± 0.002^a^	226 ± 6.9^a^	1.44 ± 0.2^a^
180 DAT (April 20, 2025)				
D3	12.58 ± 2.2^a^	0.12 ± 0.003^a^	211 ± 16.7^a^	1.27 ± 0.4^a^
D6	14.54 ± 0.6^a^	0.15 ± 0.008^a^	230 ± 0.7^a^	1.63 ± 0.2^a^
D9	13.76 ± 0.8^a^	0.14 ± 0.018^a^	229 ± 8.4^a^	1.55 ± 0.3^a^

**Note:**

Data are presented as mean ± SD (*n* = 3). ^Z^Different lowercase letters within the same column indicate significant differences among treatments according to DMRT at *p* < 0.05. Pn, net photosynthetic rate; Gs, stomatal conductance; Ci, intercellular CO₂ concentration; Tr, transpiration rate.

**Table 7:** Effects of different numbers of drippers per slab on leaf pigment contents of strawberry plants at 190 DAT (April 30, 2025).

Treatments	Chlorophyll *a* (mg/g FW)	Chlorophyll *b* (mg/g FW)	Total chlorophyll (mg/g FW)	Carotenoid content (mg/g FW)
D3	2.02 ± 0.09^bZ^	0.95 ± 0.02^b^	2.97 ± 0.09^b^	0.26 ± 0.03^b^
D6	2.38 ± 0.07^a^	1.06 ± 0.04^a^	3.45 ± 0.10^a^	0.33 ± 0.01^a^
D9	2.23 ± 0.14^ab^	1.00 ± 0.06^ab^	3.23 ± 0.20^ab^	0.30 ± 0.03^ab^

**Note:**

Data are presented as mean ± SD (*n* = 3). ^Z^Different lowercase letters within the same column indicate significant differences among treatments according to DMRT at *p* < 0.05. FW, fresh weight.

### Antioxidant and antioxidant enzyme properties

**Figure 5:**
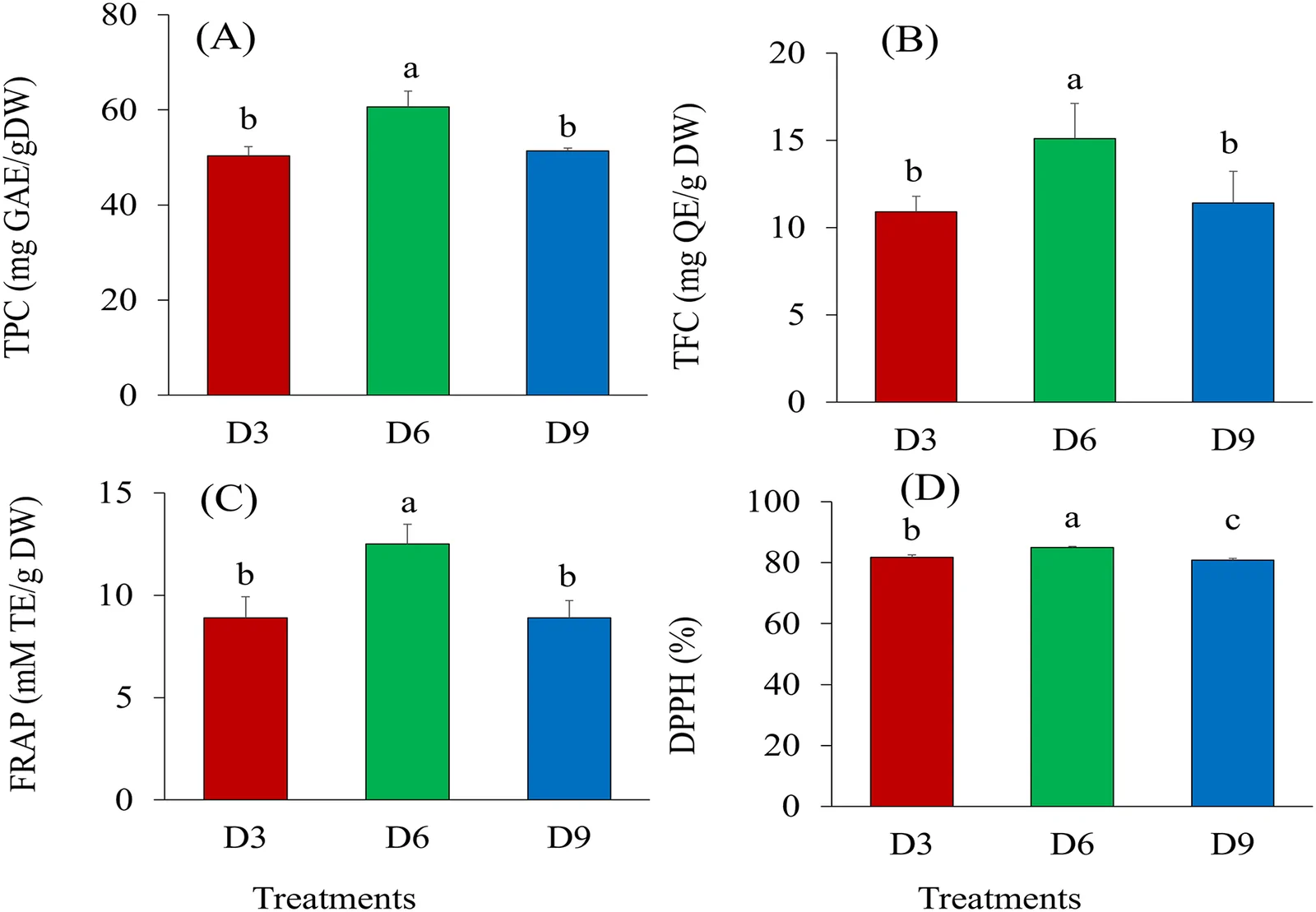
Effects of different numbers of drippers per slab on the antioxidant properties of strawberry plants at 190 DAT (April 30, 2025). (A) Total phenolic content (TPC), (B) total flavonoid content (TFC), (C) ferric reducing antioxidant power (FRAP), and (D) DPPH radical scavenging activity. Error bars represent the standard deviation (*n* = 6). Different letters above the bars indicate significant differences among treatments according to DMRT at *p* < 0.05.

**Figure 6:**
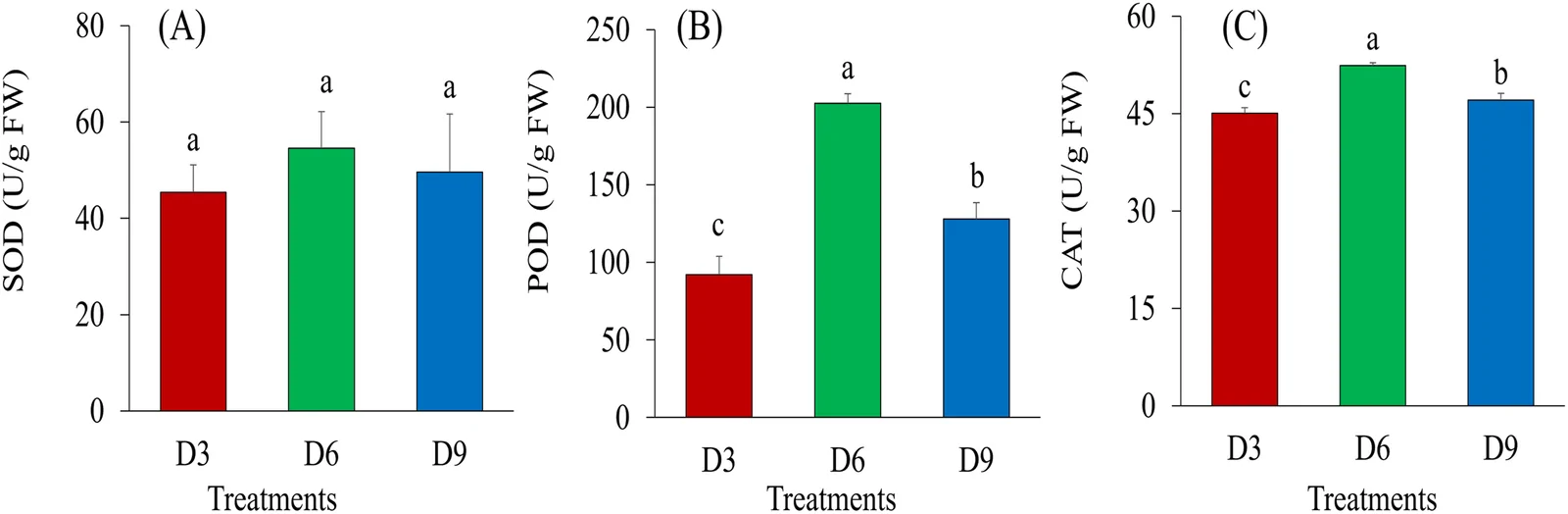
Effects of different numbers of drippers per slab on antioxidant enzyme activities in strawberry plants at 190 DAT (April 30, 2025). (A) Superoxide dismutase (SOD), (B) peroxidase (POD), and (C) catalase (CAT) activities in plants irrigated with different numbers of drippers. Error bars represent the standard deviation (*n* = 3). Different letters above the bars indicate significant differences among treatments according to Duncan’s multiple range test (DMRT) at *p* < 0.05.

As shown on Fig. 5, varying levels of drip irrigation affected TPC, TFC, FRAP, and DPPH radical scavenging activity. The D6 treatment exhibited the highest values for all antioxidant parameters: TPC (60.6 mg GAE/g DW), TFC (15.1 mg QE/g DW), FRAP (12.5 mM TE/g DW), and DPPH activity (85.0%). These values were significantly greater than those of the D3 and D9 treatments, which displayed lower ranges: TPC (50.3–51.4 mg GAE/g DW), TFC (10.9–1.4 mg QE/g DW), FRAP (8.9 mM TE/g DW), and DPPH activity (80.8–81.8%). Furthermore, antioxidant enzymes showed significant variation among the treatments (Fig. 6). SOD activity ranged from 45.4 to 54.6 U/g·FW, showing no significant variation among treatments (Fig. 6A). However, numerically the highest SOD activity was observed in D6, while the lowest was found in D3 (Fig. 6A). POD activity was highest in D6 (202.7 U/g·FW) and lowest in D3 (92.0 U/g·FW) (Fig. 6B). Similarly, CAT activity was significantly higher in D6 (52.4 U/g·FW) compared to D3 (45.1 U/g·FW) and D9 (47.1 U/g·FW) (Fig. 6C).

### Leaves mineral content

The mineral content was significantly influenced by the treatments (Table 8). Calcium (Ca) concentrations were highest in D9 (13,138 mg/kg) and D6 (12,450 mg/kg), which did not differ significantly from each other but were greater than in D3 (7,108 mg/kg). Potassium (K) content was greatest in D3 (26,409 mg/kg), followed by D6 (23,112 mg/kg) and D9 (21,647 mg/kg), with no significant difference between D6 and D9. Magnesium (Mg) content was significantly higher in D6 (4,507 mg/kg) and D9 (4,143 mg/kg) compared to D3 (3,033 mg/kg). Sodium (Na) content did not differ significantly among treatments, ranging from 1,113 to 1,185 mg/kg. Phosphorus (P) concentrations were highest in D6 (3,232 mg/kg), followed by D9 (2,562 mg/kg) and D3 (2,534 mg/kg), with no significant difference between D3 and D9. The Ca/Mg ratio ranged from 2.34 to 3.17. The lowest value was recorded in the D3 treatment (2.34), while the highest was observed in D9 (3.17). Similarly, the Ca + K + Mg/Na ratio varied between 31.45 and 35.25, with the highest value found in D6 (35.25), followed closely by D9 (35.02), and the lowest in D3 (31.45). However, no significant differences were observed among treatments for this ratio.

**Table 8:** Effects of different numbers of drippers per slab on the mineral nutrient composition of strawberry leaves at 210 DAT (May 20, 2025).

Treatments	Ca	K	Mg	Na	P	Ca/Mg	Ca + K + Mg/Na
	mg·Kg^−1^						
D3	7,108 ± 312.1^bZ^	26,409 ± 314.4^a^	3,033 ± 75.7^b^	1,185 ± 207.8^a^	2,534 ± 79.7^b^	2.34 ± 0.1^c^	31.45 ± 5.3^a^
D6	12,450 ± 860.3^a^	23,112 ± 902.5^b^	4,507 ± 413.9^a^	1,141 ± 73.6^a^	3,232 ± 212.2^a^	2.77 ± 0.1^b^	35.25 ± 3.3^a^
D9	13,138 ± 824.7^a^	21,647 ± 1534.9^b^	4,143 ± 172.2^a^	1,113 ± 54.1^a^	2,562 ± 174.0^b^	3.17 ± 0.1^a^	35.02 ± 1.9^a^

**Note:**

Data are presented as mean ± SD (*n* = 3). ^Z^Different lowercase letters within the same column indicate significant differences among treatments according to DMRT at *p* < 0.05.

### Correlation analysis among selected parameters

**Figure 7:**
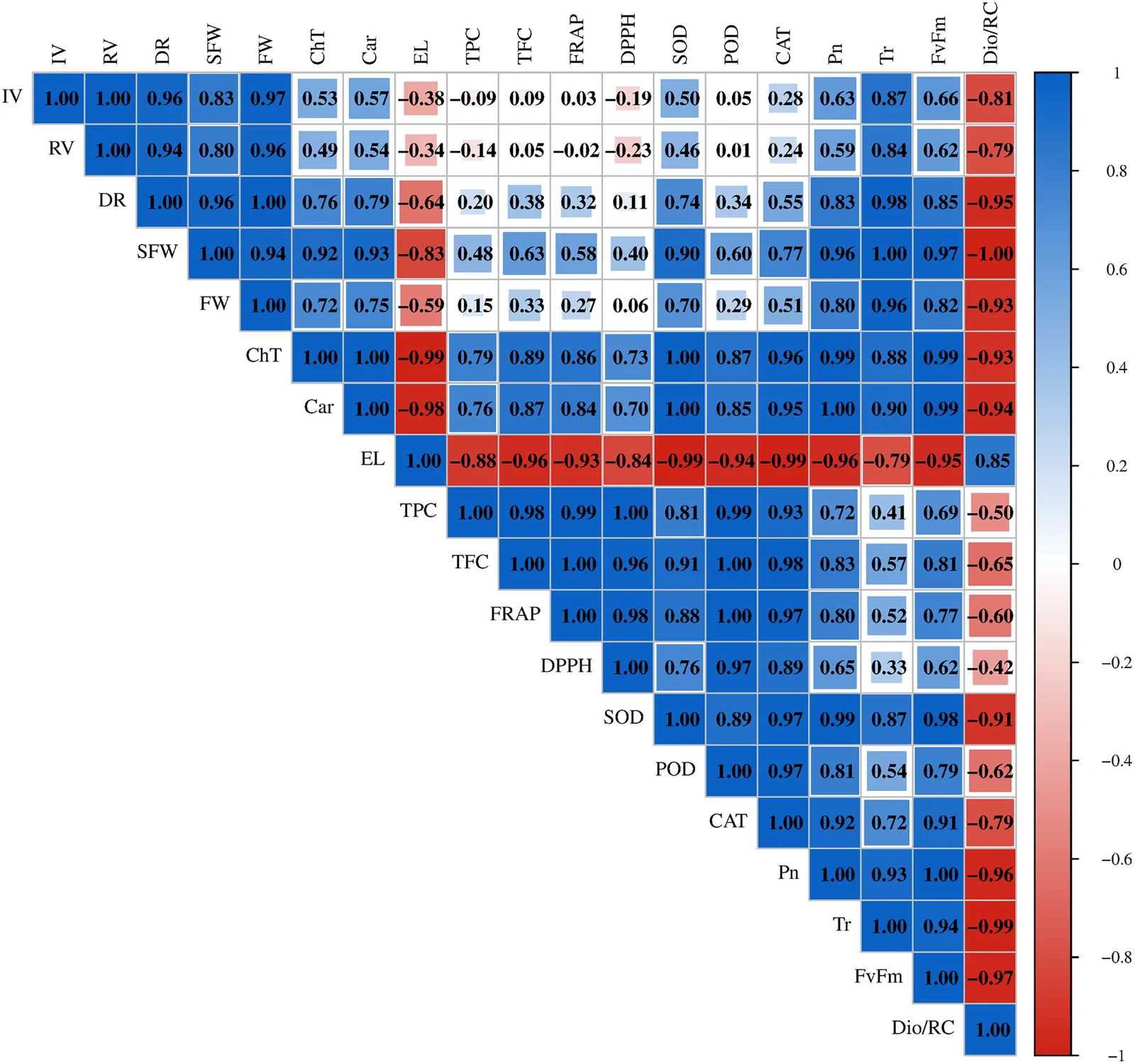
The relationship among different parameters evaluated under different drip irrigation condition. Where; IV, irrigation volume; RV, retained volume; DR, drainage rate; SFW, shoot fresh weight; TF, total fruit weight; Ch_T_, total chlorophyll content; Car, carotenoids; Fv/Fm, ratio of variable to maximum fluorescence; DIo/RC, energy dissipation per reaction center; Pn, photosynthesis rate; Tr, transpiration rate; EL, electrolytic leakage; TPC, total phenolic content; TFC, total flavonoid content; FRAP, ferric reducing antioxidant power; DPPH, 2,2-diphenyl-1-picryl-hydrazyl; SOD, superoxide dismutase; POD, peroxidase; and CAT, catalase.

Correlation analysis was conducted using parameters measured at the end of the treatment period to elucidate relationships among growth, physiological, biochemical, and antioxidant attributes under different drip irrigation conditions (Fig. 7). The analysis revealed strong positive and negative correlations among the evaluated variables, indicating functional interrelationships between water supply, plant performance, and stress responses. Irrigation volume (IV) showed a strong positive correlation with retained volume (RV) (r = 1.00) and a drainage rate (DR) (r = 0.96), suggesting that higher irrigation levels directly increased both water retention and drainage. IV also showed strong positive correlations with shoot fresh weight (SFW; r = 0.83) and fruit weight (FW; r = 0.97), demonstrating that increased irrigation enhanced biomass accumulation and fruit production. Total chlorophyll content (Ch_T_) showed strong positive correlations with SFW (r = 0.92) and FW (r = 0.72). Carotenoids (Car) also correlated positively with SFW (r = 0.93) and FW (r = 0.75), while Ch_T_ and Car were perfectly correlated (r = 1.00), indicating synchronized regulation of photosynthetic pigments under different irrigation conditions. The photosynthetic rate (Pn) was strongly correlated with Ch_T_ (r = 0.99), Car (r = 1.00), SFW (r = 0.96), and FW (r = 0.80). Transpiration rate (Tr) exhibited strong positive correlations with growth traits (SFW: r = 1.00; FW: r = 0.96) and pigments, suggesting that enhanced gas exchange contributed to improved biomass and yield. The maximum quantum efficiency of PSII (Fv/Fm) was positively correlated with SFW (r = 0.97), FW (r = 0.82), and Pn (r = 1.00), indicating improved photochemical efficiency under optimal irrigation conditions.

IV and RV showed weak negative correlations with electrolyte leakage (EL) (r ≤ –0.38). However, a comparatively stronger negative relationship was observed between DR and EL (r = –0.64). On the other hand, very weak associations were observed between irrigation- and drainage-related characteristics and antioxidant properties. Moreover, EL showed strong negative correlations with growth traits (SFW: r = –0.83; FW: r = –0.59), pigment contents (ChT: r = –0.99; Car: r = –0.98), and photosynthetic rate (Pn: r = –0.96), indicating that increased membrane damage under reduced irrigation adversely affected photosynthetic efficiency and plant productivity. Energy dissipation per reaction center (DIo/RC) was also strongly negatively correlated with growth (SFW: r = –1.00; FW: r = –0.93), pigment contents (ChT: r = –0.93; Car: r = –0.94), Pn (r = –0.99), and Fv/Fm (r = –0.97), suggesting that increased energy dissipation was associated with stress conditions and reduced photochemical efficiency. Total phenolic content (TPC), total flavonoid content (TFC), ferric reducing antioxidant power (FRAP), and DPPH radical scavenging activity showed strong positive correlations with each other (r = 0.96–1.00), indicating a coordinated enhancement of non-enzymatic antioxidant systems. These parameters were negatively correlated with EL, suggesting that enhanced antioxidant capacity helped mitigate membrane damage. Enzymatic antioxidants, including superoxide dismutase (SOD), peroxidase (POD), and catalase (CAT), also showed strong positive intercorrelations (r = 0.89–0.97) and were positively associated with TPC, TFC, FRAP, and DPPH, indicating an integrated antioxidant defense mechanism. However, SOD, POD, and CAT exhibited negative correlations with EL (r ≈ –0.99) and DIo/RC (r ranging from –0.79 to –0.96), suggesting that increased oxidative stress was accompanied by enhanced activation of enzymatic antioxidant defenses.

### Cluster analysis

**Figure 8:**
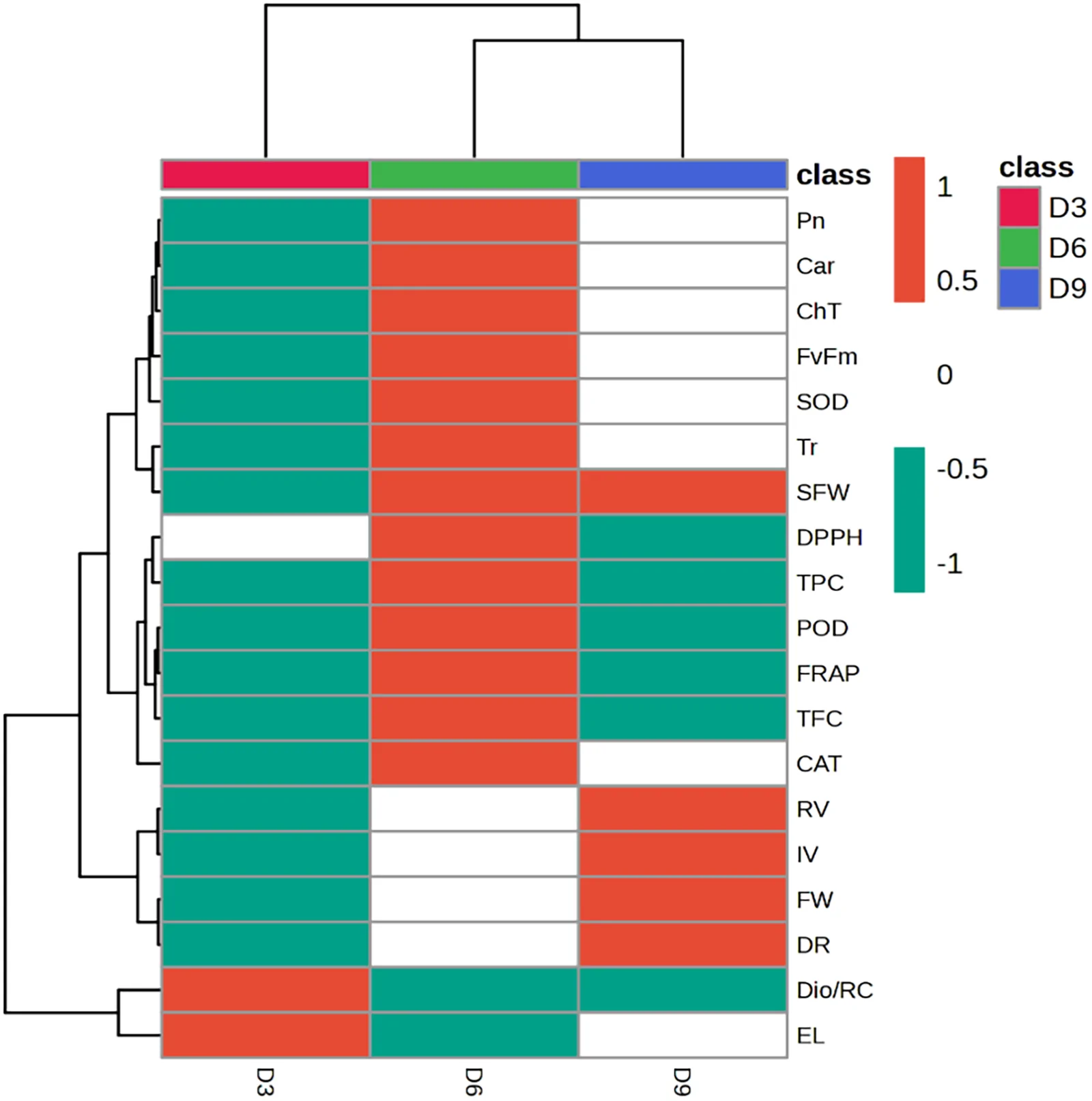
Hierarchical clustering heatmap of morphological, physiological, biochemical, and fruit quality traits of plants irrigated with different number drippers. Where; red and green colors represent higher and lower relative values, respectively, while clustering reveals treatment-specific trait associations. IV, irrigation volume; RV, retained volume; DR, drainage rate; SFW, shoot fresh weight; TF, total fruit weight; ChT, total chlorophyll content; Car, carotenoids; Fv/Fm, ratio of variable to maximum fluorescence; DIo/RC, energy dissipation per reaction center; Pn, photosynthesis rate; Tr, transpiration rate; EL, electrolytic leakage; TPC, total phenolic content; TFC, total flavonoid content; FRAP, ferric reducing antioxidant power; DPPH, 2,2-diphenyl-1-picryl-hydrazyl; SOD, superoxide dismutase; POD, peroxidase; and CAT, catalase.

To better understand treatment-specific trait associations, cluster analysis was performed. As shown in Fig. 8, the hierarchical clustering heatmap revealed clear treatment-specific patterns among morphological, physiological, biochemical, and fruit traits under different numbers of drippers. The clustering dendrogram grouped D6 and D9 more closely together, while D3 formed a relatively distinct cluster, indicating a different physiological and biochemical response under the three-dripper treatment. Most physiological traits, Pn, Car, Ch_T_, Fv/Fm, SOD, and Tr, exhibited higher relative values under the D6 treatment, whereas lower values were generally observed under D3. The D9 treatment showed intermediate values for these parameters, suggesting moderate physiological performance compared with D6. Similarly, major antioxidant-related biochemical traits such as DPPH radical scavenging activity, TPC, POD, FRAP, TFC, and CAT were predominantly elevated under D6, while reduced levels were observed under D3 and, to some extent, under D9. In contrast, water-related and growth traits, including RV, IV, FW, and DR, showed higher relative values under the D9 treatment, whereas these parameters were generally lower under D3. SFW also tended to be higher in D9 and D6 compared with D3. Conversely, stress-related indicators such DIo/RC and EL were higher under the D3 treatment, suggesting greater physiological stress when plants were irrigated with fewer drippers. These traits were lower under D6 and D9.

## Discussion

Irrigation management is crucial for strawberry cultivation as it directly impacts substrate moisture, nutrient uptake, plant health, yield, and fruit quality. Key factors such as drainage volume, rate, and retained volume indicate the efficiency of water use, the risk of nutrient leaching, and the substrate’s capacity to hold water for plant availability. Additionally, drainage pH and EC reflect nutrient dynamics; excess irrigation can alter root-zone chemistry, affecting mineral absorption and fruit composition. This study found that irrigation treatments significantly impacted drainage characteristics (Table 1). Specifically, treatment D9 exhibited higher drainage volume and rate but also higher drainage EC and lower pH, particularly during early growth stages. Although nutrient uptake increases during the vegetative growth period, the high nutrient supply appears to have influenced the pH and EC of the drainage. In contrast, treatment D3 showed lower values, indicating low water availability, which may limit nutrient mobility while enhancing EC at later stages. The observed decline in drainage volume and rate with plant age suggests improved water use efficiency and increased canopy demand, while the rise in retained volume indicates substrate stabilization as crops mature.

Growth traits, such as the number of leaves, plant height, petiole length, and biomass, indicate plant vigor and photosynthetic capacity. Enhanced growth generally correlates with higher yield potential. Adequate irrigation supports cell expansion and growth, whereas insufficient or excessive irrigation can restrict photosynthesis. Crown diameter in strawberries is crucial as it influences the plant‘s ability to support multiple inflorescences and fruit clusters. In this study, during the early growth stage (15 DAT), no significant differences were observed in most growth-related parameters (data not shown). This suggests that the treatments had little to no effect on plant growth at this stage. However, as growth progressed, the effects of the treatments became more pronounced. For instance, at 145 DAT, treatment D3 was significantly different from D6 and D9, showing the lowest values for most growth parameters (data not shown). This trend continued until the end of the experiment, as showed in Table 2. At 210 DAT, treatments D6 and D9 consistently promoted higher growth compared to D3. The higher leaf number, plant height, and petiole/leaf dimensions observed under D6 and D9 suggest improved water status and carbon assimilation. Although crown diameter was numerically higher under D6, it declined under D3 and D9, indicating that varying water availability may reduce mechanical tissue development in the crown. These results align with reports that moderate irrigation enhances strawberry vegetative growth, while both deficit and excess water can be detrimental (Yuan, Sun & Nishiyama, 2004; Ariza et al., 2021). Earlier research showed that high irrigation levels reduce crown growth, increasing susceptibility to stress (Marcellini et al., 2022). Thus, optimal water availability supports the structural and functional development of strawberry plants; however, tolerance to low and high-water stress varies among strawberry cultivars (Celiktopuz, 2023).

Fruit traits such as size, firmness, SSC, TA, and the SSC/TA ratio are critical for market quality and consumer acceptance. Firmness aids in postharvest handling, while SSC and the SSC/TA ratio influence sweetness perception and flavor balance. The D6 treatment produced the largest and widest fruits, indicating that moderate irrigation maximizes assimilate supply and cell enlargement (Table 3). Although differences in firmness were not statistically significant, D3 fruits were numerically firmer, likely due to reduced water influx and denser cell walls. SSC remained stable across treatments, but TA was higher in D3, resulting in the lowest SSC/TA ratio. Consequently, fruits from D6 and D9 were sweeter, whereas D3 fruits were more acidic. Earlier reports indicate that deficit irrigation often leads to smaller but firmer fruits with higher acidity, which is consistent with our findings for D3. In contrast, D6 aligns with research suggesting that a balanced water supply enhances fruit size and sweetness without compromising firmness (Li et al., 2024). Additionally, yield and marketable fruit weight are key economic outcomes of irrigation practices. Adequate irrigation supports fruit set, reduces the number of unmarketable fruits, and influences size distribution.

In this work, dipper density significantly influenced fruit number and fruit weight, particularly at the later stages of fruit development. At the earliest harvesting period (December–January), treatment effects on fruit number and total yield were negligible (data not shown). However, as harvesting progressed into February, March, and April, significant differences among treatments became evident. During these later harvests, the D6 and D9 treatments consistently produced the highest total fruit number and fruit weight, while the D3 treatment showed the lowest values (data not shown). Moreover, the number and weight of unmarketable fruits were significantly reduced under D6 and D9, whereas D3 resulted in the highest proportion of unmarketable yield (data not shown). This suggests that a reduced number of drippers restricted the water supply, limiting plant growth and fruit development. The overall results demonstrate that increasing the number of drippers enhanced both fruit yield and quality (Table 4). The D9 treatment produced the greatest number and weight of marketable fruits, while D6 favored the development of larger fruits (Table 4). In contrast, D3 consistently resulted in fewer and lighter fruits (Table 4). These findings highlight the importance of adequate irrigation uniformity and supply in optimizing both fruit quantity and quality. Previous studies have similarly reported that moderate irrigation increases the proportion of marketable fruits, while water stress reduces berry size and yield (Weber et al., 2017). Moreover, the higher WUE observed in D6 compared with D9 suggests that moderate drip irrigation enhanced water productivity in strawberry plants by maintaining a favorable balance between water availability and root-zone aeration, thereby improving biomass partitioning under optimized irrigation scheduling (Sim et al., 2025). In contrast, excessive irrigation significantly reduces irrigation water use efficiency due to inefficient utilization of applied water in the rhizosphere (Yuan, Sun & Nishiyama, 2004).

Physiological indicators such as LRWC, EL, gas exchange parameters, and chlorophyll fluorescence provide insights into plant water status, membrane integrity, photosynthetic performance, and stress responses. LRWC reflects the plant’s ability to maintain hydration, while EL indicates membrane damage under stress. Gas exchange traits directly measure photosynthetic capacity and stomatal regulation, whereas OJIP chlorophyll fluorescence parameters reveal PSII efficiency and energy partitioning under stress conditions. Pigment contents, including chlorophyll and carotenoids, are associated with light harvesting and photoprotection. In this study, LRWC was not significantly different among treatments, but D6 and D9 maintained higher values than D3 (Fig. 4A), indicating better hydration under adequate irrigation. EL was lowest in D6 (Fig. 4B), demonstrating improved membrane stability, while D3 exhibited higher EL, suggesting stress-induced injury. No significant differences were observed among OJIP chlorophyll fluorescence parameters at early growth stages (Table 5), indicating comparable photochemical efficiency across varying irrigation levels. However, at later growth stages, D3 plants showed significantly reduced Fm/Fo, Fv/Fo, Fv/Fm, and PiAbs, alongside increased DIo/RC, indicating long-lasting photoinhibition under water deficit. D6 maintained the highest PiAbs, reflecting better long-term photosynthetic stability at 190 DAT (Table 5). This finding aligns with Arief et al. (2022), who reported that water deficit reduces PSII efficiency and increases non-photochemical quenching in strawberries. Similar declines in PiAbs under drought conditions were observed in strawberries by Kapur et al. (2018) and Song et al. (2023), supporting the notion that irrigation alleviates photoinhibitory damage. Gas exchange parameters demonstrate the physiological capacity of leaves to fix CO₂ and regulate transpiration. At 70 DAT, D6 showed significantly higher rates of photosynthesis, stomatal conductance, intercellular CO₂ concentration, and transpiration rate compared to D3, indicating greater stomatal opening (Table 6). These differences diminished by 180 DAT, possibly due to the plants’ developmental stage. Chlorophyll *a*, *b*, and carotenoids were highest under D6, suggesting optimized photosynthetic machinery (Table 7). Correlations confirmed strong linkages between gas exchange traits, pigments, and Fv/Fm, underscoring the role of water availability in maintaining photosynthesis (Fig. 7). Similar results have been documented in Tuscan kale (He et al., 2023) and jujube trees (Qiang et al., 2025), where moderate irrigation enhances photosynthesis and pigment retention, while deficit irrigation reduces photosynthetic capacity (Fig. 7). The reduced pigments observed under D3 align with earlier studies linking water stress to chlorophyll degradation and oxidative stress (Zahedi et al., 2023).

Plants under stress accumulate ROS, which can damage cellular structures (Sood, 2025). Antioxidant metabolites and enzymes are essential for mitigating oxidative stress caused by suboptimal water conditions. A higher antioxidant capacity enhances plant resilience and contributes to the nutraceutical quality of fruit (Jomova et al., 2024). D6 treatment maximized both non-enzymatic antioxidants and enzymatic activities (Figs. 4 and 5), demonstrating that moderate irrigation promotes a balanced oxidative status in strawberries. In contrast, D3 treatment resulted in lower antioxidant defense, while D9 treatment also led to reduced antioxidant capacity, indicating that both water deficit and excessive disrupt redox homeostasis. The strong negative correlation between EL and antioxidant traits confirms that membrane damage under stress is mitigated by stronger antioxidant defenses, as observed in the D6 treatment. Similar findings were reported by Raffaelli et al. (2025) in strawberries and Malejane et al. (2018) in lettuce, who noted that moderate irrigation enhances phenolic content, while severe water deficit impairs antioxidant defense. The stimulation of POD and CAT in D6 aligns with earlier studies in strawberries, where optimal irrigation upregulated enzymatic antioxidant activity (Sun et al., 2015).

Leaf mineral composition influences growth, photosynthesis, and fruit quality. Ca and Mg are vital for structural stability and chlorophyll synthesis, while K regulates osmotic balance and sugar transport. P supports energy metabolism and growth. Higher concentrations of Ca, Mg, and P under D6 and D9 indicate that adequate irrigation enhances nutrient uptake. K was highest in D3, likely due to concentration effects from reduced water availability. The Ca/Mg ratio and Ca + K + Mg/Na ratio are important indicators of ionic balance in plant tissues, influencing nutrient uptake, membrane stability, and overall physiological performance under varying irrigation conditions. In this study, the lowest value of Ca/Mg ratio recorded under the D3 treatment and the highest under D9. This variation suggests that irrigation amount influenced the relative availability and uptake of Ca and Mg ions. A lower ratio, as observed in D3, might indicate reduced Ca mobility or greater Mg accumulation due to limited water availability (Table 8), which can restrict Ca transport through the xylem. Conversely, the higher ratio under D9 may reflect improved Ca uptake under higher irrigation levels, as adequate substrate moisture facilitates nutrient diffusion and mass flow. Similarly, the Ca + K + Mg/Na ratio, which represents the overall balance between beneficial cations (Ca, K, Mg) and Na. Although the differences among treatments were not statistically significant, the trend indicates that D6 and D9 tended to maintain a more favorable ionic balance by reducing Na accumulation or enhancing the uptake of essential cations. The absence of significant differences in the Ca + K + Mg/Na ratio among treatments suggests that the irrigation levels used in this study did not induce substantial ionic stress or nutrient imbalance. These results indicate that while variations in irrigation amount can slightly modify the ionic composition, the drip irrigation management applied here maintained a relatively stable cationic balance, which is beneficial for sustaining optimal physiological conditions and nutrient status in plants.

## Conclusions

This study evaluated the effects of different drip irrigation levels on the strawberry variety ‘Kuemsil’ in closed hydroponic system. We assessed growth, fruit yield, and various physiological, biochemical, and stress-related parameters. The highest leaf number, plant height, petiole diameter, and leaf length were observed under D6 and D9 treatments, while the lowest values were recorded under D3. Crown diameter was slightly higher in plants treated with D6. The number of marketable fruits and fruit weight peaked under D9, whereas D6 had the lowest unmarketable fruit number and weight. The largest fruit size and weight were also associated with the D6 treatment. Physiological performance varied across growth stages. Initially, no significant differences in chlorophyll fluorescence were detected; however, significant differences arose as the growth period progressed. Fm/Fo, Fv/Fo, Fv/Fm, and PiAbs were higher in D6 and D9, while ABS/RC and DIo/RC were higher in D3. Gas exchange parameters consistently showed the highest values in D6 and D9. Moreover, D6-treated plants showed the lowest EL and the highest LRWC. Improved pigment concentrations, along with enhanced antioxidant properties and enzyme activities, were recorded under D6. The results indicate that both D6 and D9 drip irrigation levels enhance growth and physiological performance in ‘Kuemsil’ strawberries. However, D6 treatment demonstrated a superior balance between fruit quality, physiological stability, and biochemical resilience, making it the most effective irrigation level for optimizing yield and fruit quality while minimizing stress damage. It is recommended that strawberry growers adopt the D6 drip irrigation level for cultivating ‘Kuemsil’ under similar conditions, as it maximizes marketable yield, improves fruit size and antioxidant properties, and maintains plant physiological health.

## Supplemental Information

10.7717/peerj.21637/supp-1 Supplemental Information 1 Raw data used in the current study.

## Additional Information and Declarations

### Data Availability

The following information was supplied regarding data availability:

The raw measurements are available in the Supplemental File.

## References

[ref-35] Arief MA, Kim H, Kurniawan H, Nugroho AP, Kim T, Cho BK (2022). Chlorophyll fluorescence imaging for early detection of drought and heat stress in strawberry plants. Plants.

[ref-1] Ariza MT, Miranda L, Gómez-Mora JA, Medina JJ, Lozano D, Gavilán P, Soria C, Martínez-Ferri E (2021). Yield and fruit quality of strawberry cultivars under different irrigation regimes. Agronomy.

[ref-2] Asaduzzaman M, Niu G, Asao T (2022). Nutrients recycling in hydroponics: opportunities and challenges toward sustainable crop production under controlled environment agriculture. Frontiers in Plant Science.

[ref-3] Blanke MM, Cooke DT (2004). Effects of flooding and drought on stomatal activity, transpiration, photosynthesis, water potential and water channel activity in strawberry stolons and leaves. Plant Growth Regulation.

[ref-4] Brand-Williams W, Cuvelier ME, Berset CLWT (1995). Use of a free radical method to evaluate antioxidant activity. LWT-Food Science and Technology.

[ref-5] Celiktopuz E (2023). Determination of drought tolerance of different strawberry genotypes. PeerJ.

[ref-6] Chang CC, Yang MH, Wen HM, Chern JC (2002). Estimation of total flavonoid content in propolis by two complementary colorimetric methods. Journal of Food and Drug Analysis.

[ref-7] Daniel K, Hartman S (2024). How plant roots respond to waterlogging. Journal of Experimental Botany.

[ref-8] Dutta S, Mukherjee B, Sawarkar A (2023). Enhanced agricultural productivity using hydroponics technique: a smart farming system. Irrigation Systems and Applications.

[ref-9] He J, Chang C, Qin L, Lai CH (2023). Impacts of deficit irrigation on photosynthetic performance, productivity and nutritional quality of aeroponically grown Tuscan Kale (Brassica oleracea L.) in a tropical greenhouse. International Journal of Molecular Sciences.

[ref-10] Jomova K, Alomar SY, Alwasel SH, Nepovimova E, Kuca K, Valko M (2024). Several lines of antioxidant defense against oxidative stress: antioxidant enzymes, nanomaterials with multiple enzyme-mimicking activities, and low-molecular-weight antioxidants. Archives of Toxicology.

[ref-11] Kapur B, Çeliktopuz E, Sarıdaş MA, Paydaş KS (2018). Irrigation regimes and bio-stimulant application effects on yield and morpho-physiological responses of strawberry. Horticultural Science and Technology.

[ref-12] Kim DH, Shawon MRA, An JH, Lee HJ, Lee Y-J, Kim M, Lee Y-B, Choi KY (2022). Effects of drip irrigation volumes on plant growth and yield of tomato grown in perlite. Journal of Bio-Environment Control.

[ref-13] Li D, Li M, Yang X, Chen J, Zhang Z (2024). Optimal use of irrigation water and fertilizer for strawberry based on weighing production benefits and soil environment. Irrigation Science.

[ref-14] Malejane DN, Tinyani P, Soundy P, Sultanbawa Y, Sivakumar D (2018). Deficit irrigation improves phenolic content and antioxidant activity in leafy lettuce varieties. Food Science & Nutrition.

[ref-15] Mao W, Zhu Y, Wu J, Ye M, Yang J (2022). Evaluation of effects of limited irrigation on regional-scale water movement and salt accumulation in arid agricultural areas. Agricultural Water Management.

[ref-16] Marcellini M, Mazzoni L, Raffaelli D, Pergolotti V, Balducci F, Capocasa F, Mezzetti B (2022). Evaluation of single-cropping under reduced water supply in strawberry cultivation. Agronomy.

[ref-18] Qiang W, Ai P, Ma Y, Zhao J (2025). The impact of water deficit at various growth stages on physiological characteristics, fruit yield, and quality of drip-irrigated jujube trees. Agronomy.

[ref-19] Raffaelli D, Qaderi R, Mazzoni L, Mezzetti B, Capocasa F (2025). Yield and sensorial and nutritional quality of strawberry (*Fragaria× ananassa* Duch.) fruits from plants grown under different amounts of irrigation in soilless cultivation. Plants.

[ref-20] Sim HS, Jo JS, Moon YH, Jung SB, Lee TY, Shin HR, Kim YJ, Kim NK, Kim SK (2025). Development of a strawberry transpiration model based on a simplified Penman-Monteith model under different irrigation regimes. Horticultural Environment and Biotechnology.

[ref-21] Song Y, Kim CW, Kweon H, Yoon KK, Shim YJ, Lee UK, Lee KC (2023). Effects of drought stress on the growth and physiological characteristics of grafted Ziziphus jujuba var. inermis under RCP 6.0 climate change scenarios. Horticultural Science and Technology.

[ref-22] Sood M (2025). Reactive oxygen species (ROS): plant perspectives on oxidative signalling and biotic stress response. Discover Plants.

[ref-23] Sun C, Li X, Hu Y, Zhao P, Xu T, Sun J, Gao X (2015). Proline, sugars, and antioxidant enzymes respond to drought stress in the leaves of strawberry plants. Horticultural Science and Technology.

[ref-24] Weber N, Zupanc V, Jakopic J, Veberic R, Mikulic-Petkovsek M, Stampar F (2017). Influence of deficit irrigation on strawberry (*Fragaria× ananassa* Duch.) fruit quality. Journal of the Science of Food and Agriculture.

[ref-25] Yi H, Chen Y, Anderson CT (2022). Turgor pressure change in stomatal guard cells arises from interactions between water influx and mechanical responses of their cell walls. Quantitative Plant Biology.

[ref-26] Yuan BZ, Sun J, Nishiyama S (2004). Effect of drip irrigation on strawberry growth and yield inside a plastic greenhouse. Biosystems Engineering.

[ref-27] Zahedi SM, Hosseini MS, Fahadi Hoveizeh N, Kadkhodaei S, Vaculík M (2023). Physiological and biochemical responses of commercial strawberry cultivars under optimal and drought stress. Plants.

[ref-28] Zebro M, Baek J, Kim M, Jeong Y, Rabbani MG, Choi K (2025). Nutrient solution strength affects growth, physiology, biochemistry and fruit quality of Korean strawberry ‘Kuemsil’ in a recycling hydroponic system. Frontiers in Plant Science.

[ref-29] Zebro M, Heo JY (2024a). Salicylic acid treatment improves the shelf life and quality of ‘Cheonghyang’ grapes during cold storage. South African Journal of Enology and Viticulture.

[ref-30] Zebro M, Heo JY (2024b). Rootstock type differentially affects pollen germination characteristics and chilling stress response at flowering time in ‘Fuji’ apple. Applied Fruit Science.

[ref-31] Zhao W, Liu L, Shen Q, Yang J, Han X, Tian F, Wu J (2020). Effects of water stress on photosynthesis, yield, and water use efficiency in winter wheat. Water.

[ref-32] Zhou C, Zhang H, Yu S, Chen X, Li F, Wang Y, Wang Y, Liu L (2023). Optimizing water and nitrogen management strategies to improve their use efficiency, eggplant yield and fruit quality. Frontiers in Plant Science.

